# Efficacy and Safety of Intravesical OnabotulinumtoxinA Injection on Elderly Patients with Chronic Central Nervous System Lesions and Overactive Bladder

**DOI:** 10.1371/journal.pone.0105989

**Published:** 2014-08-22

**Authors:** Yuan-Hong Jiang, Chun-Hou Liao, Dong-Ling Tang, Hann-Chorng Kuo

**Affiliations:** 1 Department of Urology, Buddhist Tzu Chi General Hospital and Tzu Chi University, Hualien, Taiwan; 2 Department of Urology, Cardinal Tien Hospital and School of Medicine, Fu-Jen Catholic University, New Taipei, Taiwan; Carolina Urologic Research Center, United States of America

## Abstract

**Purpose:**

Intravesical injection of onabotulinumtoxinA is an effective treatment for overactive bladder (OAB). Nonetheless, the treatment outcome is unclear in OAB patients with central nervous system (CNS) lesions. This study evaluated the efficacy and safety of intravesical onabotulinumtoxinA treatment in elderly patients with chronic cerebrovascular accidents (CVAs), Parkinson’s disease (PD) and dementia.

**Materials and Methods:**

Patients with CVA, PD, dementia, and OAB refractory to antimuscarinic therapy were consecutively enrolled in the study group. Age-matched OAB patients without CNS lesions were selected to serve as a control group. OnabotulinumtoxinA (100 U) was injected into the bladder suburothelium at 20 sites. The clinical effects, adverse events, and urodynamic parameters were assessed at baseline and 3 months post-treatment. The Kaplan-Meier method was used to compare long-term success rates between groups.

**Results:**

A total of 40 patients with OAB due to CVA (23), PD (9), dementia (8) and 160 control patients were included in this retrospetive analysis. Improvement of urgency severity scale, increased bladder capacity and increased post-void residual volume were comparable between the groups at 3 months. Patients with CNS lesions did not experience increased risks of acute urinary retention and urinary tract infection; nonetheless, patients with CVA experienced a higher rate of straining to void. Long-term success rates did not differ between the patients with and without CNS lesions.

**Conclusion:**

Intravesical injection of 100 U of onabotulinumtoxinA effectively decreased urgency symptoms in elderly OAB patients with CNS lesions. The adverse events were acceptable, and long-term effects were comparable to OAB patients in general. Nonetheless, the possibility of longstanding urinary retention and chronic catheterization need careful evaluation for this very vulnerable population before choosing intravesical onabotulinumtoxinA treatment.

## Introduction

OAB is highly prevalent in elderly patients and involves both peripheral and CNS factors [1]. The incidence of OAB increases with age, especially in patients with CNS disorders such as CVA and PD. White matter disease causing dementia increases significantly with age and can also cause OAB and urinary incontinence [2]. The reported incidence of urinary incontinence varied from 33% to 79% in patients with CVA [3], 33.1% with PD, and 50.9% with multiple sclerosis [4]. Most of the patients with CNS disorders and OAB had NDO.

The incidence of OAB increases with aging; thus, degeneration of the CNS in the elderly is proposed as one of the pathogenic factors of OAB [1]. In patients older than 60 years with irritative urinary symptoms, brain magnetic resonance imaging showed subclinical high-intensity ischemic changes in basal ganglia in 82.6% of elderly OAB patients. Thus, aging and CNS lesions play key roles in the pathogenesis of OAB among elderly patients [5]. Both storage and voiding symptoms occur in patients with CNS disorders, resulting in a complex combination of complaints [6]. The HR-QoL in patients with CNS lesions and OAB is worse than in patients with OAB in general [7].

Antimuscarinic agents are the predominant pharmacological treatment for patients with OAB [8]. Although antimuscarinic treatment has a high success rate, cognitive dysfunction during treatment with nonselective antimuscarinic agents for OAB is of growing concern [9]. In the recent decade, intravesical injection of BoNT-A emerged as an effective treatment for OAB among patients refractory or intolerable to antimuscarinic agents [10]. BoNT-A significantly improves OAB symptoms and urodynamic parameters in NDO and OAB. However, increased PVR volume and risk of UTI after BoNT-A treatment remain concerns among frail elderly patients [11].

Although intravesical BoNT-A injections for patients with NDO due to spinal cord injury or idiopathic OAB have been extensively investigated, data on BoNT-A treatment for patients with CNS lesion such as CVA, PD, and dementia are rare. Because CNS lesions usually occur in elderly patients and their bladder symptoms are more complicated to manage than those caused by OAB in general, intravesical BoNT-A treatment might not be as effective and safe as in other OAB patients. This study evaluated the efficacy and safety of intravesical onabotulinumtoxinA (100 U) treatment on patients with chronic CNS lesions and OAB due to CVA, PD, or dementia.

## Methods

This study encompassed a retrospective analysis of therapeutic effects and adverse events in elderly patients with CNS lesions and OAB. Because this was a retrospective study, we could not enroll patients based on a power calculation. Patients with chronic CVA, PD, dementia, and OAB refractory to antimuscarinic therapy were consecutively enrolled in the study group from 2005 through 2012. These studies have been approved by the institutional review board of the Buddhist Tzu Chi General Hospital, No: 094-08, 095-73 and 095-10. Written informed consent was given by participants for their clinical records to be used in this study.

The patients with CNS lesion were diagnosed by neurologists and regularly treated at the hospital’s neurology department. OAB patients without CNS lesions treated during the same period were selected as a control group. The OAB patients included in the study were older than 60 years of age and were selected from previous clinical trials at the hospital [12]–[15]. The institutional review board and ethics committee of the hospital approved the studies. All patients were informed about possible adverse events related to onabotulinumtoxinA injection and gave written, informed consent before treatment.

The inclusion criteria were OAB symptoms with or without urinary incontinence refractory to previous behavioral modification and antimuscarinic therapy for more than 3 months. Patients with CVA, PD, and dementia had to be ambulatory, able to communicate, and record a voiding diary. Exclusion criteria were inability to ambulate (bed-ridden or completely wheel chair bound), acute or chronic urinary tract infection, presence of bladder outlet obstruction, abnormal liver function, elevated serum creatinine, and PVR volume >150 ml at enrollment.

Patients received suburothelial injections of onabotulinumtoxinA (Allergan, Irvine, CA, USA) at 20 sites using a 23-gauge needle in a rigid cystoscopic injection instrument (22 Fr, Richard-Wolf, Knittlingen, Germany). One hundred units of onabotulinumtoxinA were reconstituted to 10 ml with normal saline. The injection sites were equally distributed on the lateral and posterior bladder walls, and 0.5 ml (5 U) was injected into the suburothelial space at each site, sparing the trigone. During the injections, the bladder volume was kept at 100–150 ml and injection into blood vessels was avoided.

All procedures were performed under intravenous general anesthesia in the operating room. Anticoagulant therapy was discontinued 1 week before onabotulinumtoxinA injection. A 14-Fr Foley indwelling catheter was inserted overnight, and the patients were discharged the next morning. Broad-spectrum prophylactic antibiotics were given postoperatively for 3 days. Patients who developed acute urinary retention or PVR volumes greater than 250 ml were advised to perform clean intermittent catheterization (CIC) or clean intermittent self-catheterization (CISC) until the PVR decreased to less than 200 ml.

All patients were closely monitored every month after onabotulinumtoxinA injection until the response to onabotulinumtoxinA had disappeared. Urgency episodes and UUI were verified using a 3-day voiding diary. A validated USS questionnaire was used to grade the severity of urgency, which was linguistically translated from the validated Patients Perception of Intensity of Urgency Scale [16]. The USS graded urgency as 0, 1, 2, 3, or 4 corresponding to no feeling of urgency, mild, moderate, severe feeling of urgency, and inability to hold urine, respectively [17]. After each visit, all patients graded the treatment outcome of onabotulinumtoxinA injection based on changes to the USS. An improvement in USS by ≥1 was considered successful treatment. Any adverse event considered possibly related to the onabotulinumtoxinA treatment was recorded. These events included acute urinary retention, hematuria, general weakness, large PVR, straining to void, and UTI during the follow-up period.

Patients routinely underwent VUDS at baseline for diagnosis of DO and detection of BOO and 3 months after treatment. The parameters included CBC, Qmax, Pdet.Qmax, and PVR volume. VUDS was performed and terminology was defined according to the standards of the International Continence Society [18].

Parametric, continuous data are expressed as means and standard deviations while categorical data are expressed as numbers and percentages. Mean values of continuous variables were compared using the Mann-Whitney U-test, whereas categorical variables were compared using the Fisher exact test. Kaplan-Meier survival plots were constructed to analyze the cumulative success rates with time among groups. A p value<0.05 was considered statistically significant.

## Results

This retrospective study included 40 patients with OAB due to CNS lesions (23 with CVA, 9 with PD, 8 with dementia) and 160 OAB patients without CNS lesions. The mean age of the patients with and without CNS lesions was 74.6±7.5 and 74.0±9.3 years, respectively (p = 0.661). The mean age was comparable among the three subgroups with CNS lesions. The mean duration of CNS lesions from diagnosis was 4.3±3.1 years.

The voiding diary, USS, and urodynamic parameters at baseline did not differ significantly between the patient groups with and without CNS lesions (Table 1). Patients with and without CNS lesions experienced significant improvements of USS and UUI episodes per 3 days 3 months after onabotulinumtoxinA treatment. Urodynamic parameters also showed significant increases of CBC and PVR in both groups with and without CNS lesions. The Pdet.Qmax and Qmax did not change in patients with CNS lesions, and there was no significant difference between the groups.

**Table 1:** Changes of Voiding Diary and Urodynamic Variables after OnabotulinumtoxinA Treatment in Overactive Bladder Patients with Central Nervous System Lesion and Control Patients.

		CNS lesion	Control	P values#
		(n = 40)	(n = 160)	
**USS**	Baseline	3.72±0.67	3.68±0.64	0.777
	3 months	2.83±1.30	2.70±1.17	
	P =	0.003	<0.0001	
**Urgency**	Baseline	45.1±24.7	56.8±28.7	0.623
**per 3 days**	3 months	34.4±25.7	40.5±28.3	
	P =	0.071	0.138	
**UUI/3 days**	Baseline	12.4±12.7	19.3±18.5	0.963
	3 months	4.25±9.80	10.7±15.5	
	P =	0.010	0.035	
**Cystometric**	Baseline	220±119	248±119	0.067
**Bladder**	3 months	347±165	309±147	
**Capacity(ml)**	P =	0.001	<0.0001	
**Pdet.Qmax**	Baseline	26.5±16.0	26.8±13.7	0.996
**(cmH_2_O)**	3 months	22.1±12.6	22.3±12.6	
	P =	0.123	<0.0001	
**Qmax (ml/s)**	Baseline	9.95±4.54	13.1±7.56	0.067
	3 months	11.3±6.57	11.4±6.14	
	P =	0.353	0.019	
**PVR (ml)**	Baseline	47.9±46.0	41.4±66.4	0.214
	3 months	157±130	120±116	
	P =	<0.0001	<0.0001	

# Comparison of the changes of variables from baseline to 3 months within each group.

CNS: central nervous system, OAB: overactive bladder, USS: urgency severity score, UUI: urgency urinary incontinence, Qmax: maximum flow rate, Pdet.Qmax: detrusor pressure at Qmax, PVR: post-void residual.

Table 2 lists the changes of voiding diary variables and urodynamic parameters after onabotulinumtoxinA treatment for each CNS lesion group and control group. Patients with PD and patients in the control group experienced significant improvement of USS. Urgency and UUI episodes also improved among CVA and control patients. CBC significantly increased in CVA, dementia, and control patients. Compared with baseline, PVR volume increased significantly 3 months after therapy in patients with CVA, PD, and the control group; however, the PVR change did not significantly different from the control group (p = 0.214).

**Table 2:** Changes of Voiding Diary and Urodynamic Variables after OnabotulinumtoxinA Treatment in Overactive Bladder Patients with Cerebrovascular Disease, Parkinson’s Disease, Dementia and Patients without Central Nervous System Lesion.

		N =	Baseline	3 months	p values#
**USS**	CVA	23	3.57±0.79	3.00±1.29	0.103
	PD	9	3.71±0.76	2.43±1.27	0.022
	Dementia	8	4.00±0.00	3.25±1.50	0.391
	Control	160	3.68±2.70	2.70±1.17	<0.0001
**Urgency**	CVA	23	36.8±24.0	28.5±27.1	0.047
**Per 3 days**	PD	9	56.0±26.4	43.0±16.3	0.329
	Dementia	8	41.3±23.3	30.5±37.5	0.513
	Control	160	56.8±28.7	40.5±28.3	0.138
**UUI/3 days**	CVA	23	13.5±12.5	5.67±1.21	0.050
	PD	9	10.8±16.8	9.67±15.0	0.749
	Dementia	8	13.3±7.85	1.50±3.00	0.087
	Control	160	19.3±18.5	10.7±15.5	0.035
**Cystometric**	CVA	23	198±108	358±162	0.002
**Bladder**	PD	9	266±121	283±181	0.780
**Capacity(ml)**	Dementia	8	202±97.8	448±89.0	0.001
	Control	160	248±119	309±147	<0.0001
**Pdet.Qmax**	CVA	23	31.0±21.8	27.3±18.2	0.334
**(cmH_2_O)**	PD	9	26.3±13.6	21.1±7.34	0.409
	Dementia	8	18.0±2.0	13.7±1.53	0.006
	Control	160	26.8±13.7	22.3±12.6	<0.0001
**Qmax**	CVA	23	9.04±4.567	12.2±6.47	0.106
**(ml/s)**	PD	9	12.1±4.81	11.6±7.83	0.872
	Dementia	8	8.75±2.50	7.33±2.01	0.109
	Control	160	13.1±7.56	11.4±6.4	0.019
**PVR**	CVA	23	56.5±53.7	169±131	0.002
**(ml)**	PD	9	36.7±32.4	114±109	0.048
	Dementia	8	37.2±35.6	194±165	0.125
	Control	160	56.5±53.7	120±116	<0.0001

# Comparison of the changes of variables from baseline to 3 months within each group. CVA: cerebrovascular accident, PD: Parkinson’s disease, USS: urgency severity score, UUI: urgency urinary incontinence, Qmax: maximum flow rate, Pdet.Qmax: detrusor pressure at Qmax, PVR: Post-void residual.

Table 3 shows the incidences of adverse events in the CVA, PD, dementia, and control groups after onabotulinumtoxinA treatment. The incidence of straining to void was significantly greater in CVA subgroup. The other adverse events such as acute urinary retention, large PVR, and UTI did not significantly differ among the groups with and without CNS lesions. No adverse CNS event related to onabotulinumtoxinA injection occurred.

**Table 3:** Success Rates and Adverse Events among Overactive Bladder Patients with Cerebrovascular Disease, Parkinson’s Disease, Dementia, and Patients without Central Nervous System Lesion.

	Age (years)	AUR	PVR >150 ml	Straining to void	Hematuria	UTI	General weakness
CVA (n = 23)	73.6±7.5	4 (17.4%)	12 (52.2%)	17 (73.9%)	2 (8.7%)	1 (4.3%)	1 (4.3%)
PD (n = 9)	73.6±11.2	1 (11.1%)	3 (33.3%)	1 (11.3%)	1 (11.1%)	2 (22.2%)	1 (11.1%)
Dementia (n = 8)	76.2±9.7	0	1 (12.5%)	2 (25.0%)	0	0	0
Control (n = 160)	74.6±7.5	16 (10%)	63 (39.3%)	81 (50.6%)	16 (10%)	22 (13.8%)	6 (3.8%)
P values	0.864	0.682	0.224	0.021	0.886	0.247	0.464

CVA: cerebrovascular accident, PD: Parkinson’s disease, AUR: acute urinary retention, PVR: post void residual, UTI: urinary tract infection.

**Figure 1:**
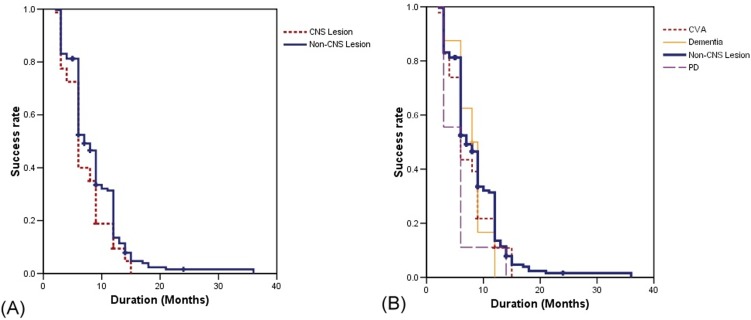
Kaplan-Meier survival curves for long-term success rates of patients with CNS lesions and the control group after intravesical onabotulinumtoxinA injection for OAB refractory to antimuscarinic therapy. (A) Success rates between OAB patients with and without CNS lesions. (B) Success rates among patients with different CNS lesions and the control group.

During the follow-up period, the therapeutic duration of onabotulinumtoxinA was similar among all subgroups with CNS lesions and the control group (Table 4). Figure 1 shows the cumulative success rates after onabotulinumtoxinA treatment between OAB patients with and without CNS lesions and among control OAB patients and subgroup patients with CNS lesions. The results show that the long-term success did not differ between patients with and without CNS lesions or among CNS lesion subgroups (all p>0.05).

**Table 4:** The Long-term Therapeutic Duration of Overactive Bladder Patients with Cerebrovascular Disease, Parkinson’s Disease, Dementia, and Patients without Central Nervous System Lesion.

Therapeutic duration				
	1–3 months	4–6 months	7–12 months	>12 months
CVA (n = 23)	4 (17.4%)	9 (39.1%)	9 (39.1%)	1 (4.3%)
PD (n = 9)	4 (44.4%)	4 (44.4%)	0	1 (11.1%)
Dementia (n = 8)	1 (12.5%)	2 (25.0%)	5 (62.5%)	0
Control (n = 160)	27 (16.9%)	53 (33.1%)	61 (38.1%)	19 (11.9%)
Total (n = 200)	3 (18.0%)	68 (34.0%)	75 (37.5%)	21 (10.5%)

CVA: cerebrovascular accident, PD: Parkinson’s disease.

## Discussion

The results showed that elderly patients with OAB due to CNS lesions such as CVA, PD, or dementia refractory to antimuscarinic therapy can be effectively treated with intravesical injection of 100 U onabotulinumtoxinA. The adverse events and incidences of acute urinary retention and UTI were comparable to OAB patients in general.

Among elderly people with CNS lesions, OAB is common. In a community health survey, 31% of patients with CNS disease reported OAB symptoms, and the overall prevalence of neurogenic OAB was 0.6%. Patients with neurogenic OAB have poorer HR-QoL compared to patients with general OAB.^7^ Among neurogenic OAB patients, CVA, PD, and multiple sclerosis are frequently encountered and cause NDO and UUI [1]. The pathogenesis of OAB in cerebral events involves not only sensory perception but also the impairment of detrusor contractility. In addition, patients with CVA or PD have impaired detrusor contractility or detrusor underactivity resulting in increased PVR volumes [19], [20]. Urethral sphincter dyssynergia, poor relaxation of the external urethral sphincter, and poor relaxation of the pelvic floor muscles are associated with DO in patients with CVA and PD [21]. Anticholinergic treatment of OAB among patients with CNS lesions could cause CNS adverse events and impaired bladder emptying.

Previous studies showed beneficial effects for 200-U onabotulinumtoxinA injections into the detrusor for patients with OAB due to PD and multiple system atrophy [22]. A recent study using 100 U onabotulinumtoxinA on patients with OAB due to PD showed similarly effective outcomes [23]. In addition, we previously reported suburothelial injections of 200 U onabotulinumtoxinA improved OAB symptoms and increased bladder volume in patients with CVA [24]. Nonetheless, PVR volume and voiding difficulty also increased after treatment. We found no clinical report of onabotulinumtoxinA effects among patients with dementia and OAB.

The pathogenesis of OAB and DO due to CNS lesions is not well known. Impaired sensory perception during bladder filling before reaching bladder capacity could play a key role in patients with OAB due to CNS lesions such as CVA, PD, and dementia. Therefore, the guarding time for these patients to prevent urinary incontinence could be shorter than that for OAB patients in general. Intravesical onabotulinumtoxinA injections decrease detrusor contractility and also modulate afferent fibers in NDO [25]. A higher dose of onabotulinumtoxinA could impair detrusor contractility and sensory input too much, causing large PVR volumes, difficult urination, or overflow incontinence. Under these considerations, a small dose of BoNT-A such as 100 U of onabotulinumtoxinA could be an optimal dose for OAB due to CNS lesions.

Intravesical injection of 100 U of onabotulinumtoxinA effectively improved OAB symptoms and voiding diary and urodynamic parameters without increasing the risks of adverse events. The long-term therapeutic effects of onabotulinumtoxinA treatment of OAB patients with CNS lesions were similar to those of OAB patients in general. These results confirm that the intravesical injection of 100 U of onabotulinumtoxinA is an effective and safe treatment option for patients with OAB due to CVA, PD, and dementia. However, most of the patients with CNS lesions are vulnerable and usually cannot handle bladder management by themselves. The risk of UTI increases when PVR increases after onabotulinumtoxinA injection, and the risk of CIC/CISC also increases [26], [27]. The recovery duration from chronic urinary retention is also significantly increased in these frail elderly patients [11].

The patients enrolled in this study were patients with mild CVA, PD or dementia who were ambulatory, able to communicate, record a voiding diary and had PVR volume <150 ml. Patient selection in this vulnerable patient population is important because patients with CNS lesions and severe physical or mental impairment might have impaired bladder sensation after intravesical onabotulinumtoxinA injection and develop chronic urinary retention and recurrent UTI. When acute urinary retention or large PVR volume develops after onabotulinumtoxinA injection, a temporary indwelling Foley catheter is mandatory to prevent overdistention of the bladder. However, if patients wish to be completely dry and avoid an indwelling catheter, a care-giver should be instructed on CIC to empty the patient’s bladder periodically. In patients who can accept and be instructed to perform CIC/CISC before onabotulinumtoxinA injection, the QoL and patient satisfaction are not affected by the need for CIC [28]. Nevertheless, the complications of UTI, possibility of chronic urinary retention, and its potential impact on health cost and burden on the caregiver still should be thoroughly discussed with patients’ families for decision making [29]. All OAB patients with CVA, PD, or dementia should be informed of the possible adverse events and bladder management strategies before institution of onabotulinumtoxinA treatment.

The main limitations of the study were the small number of patients with CNS lesions and the lack of a randomized control group for comparison. In addition, only patients with mild physical impairment were studied, which limits the application of the results to all patients with CNS lesions and OAB. Nevertheless, because intravesical onabotulinumtoxinA injection ameliorated OAB symptoms without adverse CNS events due to anticholinergic effects, we recommend it as an alternative treatment for patients with refractory OAB due to CNS lesions.

## Conclusion

The results confirmed that intravesical onabotulinumtoxinA 100 U injection effectively decreased urgency symptoms in patients with CVA, PD or dementia. The patients selected in this study had mild CNS lesions; adverse events were acceptable, and long-term effects were comparable to OAB patients in general. Nonetheless, the possibility of longstanding urinary retention and chronic catheterization need careful evaluation for this very vulnerable population before choosing intravesical onabotulinumtoxinA treatment.
